# Epidemiological analysis of paediatric tuberculosis infection in northern Saskatchewan First Nations communities, 2018–2022

**DOI:** 10.14745/ccdr.v50i12da04

**Published:** 2024-12-05

**Authors:** Nnamdi Ndubuka, Emmanuel Dankwah, Richa Tikoo, Grace Akinjobi, Tina Campbell, Tiffany Adam, Kevin Mageto, Shree Lamichhane

**Affiliations:** 1 Northern Inter-Tribal Health Authority, Prince Albert, SK; 2 School of Public Health, University of Saskatchewan, Saskatoon, SK; 3 Community Health and Epidemiology, University of Saskatchewan, Saskatoon, SK

**Keywords:** tuberculosis, paediatric tuberculosis, First Nations, northern Saskatchewan

## Abstract

**Background:**

Paediatric tuberculosis (TB), or TB in children younger than 15 years of age, is a growing public health concern in First Nations communities.

**Objective:**

To describe the epidemiology of paediatric TB in northern Saskatchewan’s on-reserve First Nations communities.

**Methods:**

We examined the paediatric TB cases reported in northern Saskatchewan First Nations on-reserve communities from 2018 to 2022 using the Northern Inter-Tribal Health Authority database. We employed descriptive statistics to understand the paediatric TB epidemiology in these susceptible populations.

**Results:**

Sixty paediatric TB cases were identified over the study period: four cases in 2018, six cases each in 2019 and 2020, 16 cases in 2021 and 28 cases in 2022. The average annual incidence was 112.6 cases per 100,000 children, ranging from 36.1 in 2018 to 268.6 in 2022. Children younger than five years of age constituted 55% of cases, with males comprising 60%. The Far North Central and East zones accounted for 90% of cases. Most cases (85%) were detected through contact tracing and pulmonary TB comprised 85% of cases. Of these, 71% completed therapy, while 27% were still in treatment. Cases were predominantly from communities with low education (100%), inadequate housing (67%) and low income (67%).

**Conclusion:**

Paediatric TB incidence among First Nations in northern Saskatchewan is increasing, especially among children younger than five years of age. Our study identifies disparities in paediatric TB incidence across demographics and geographic areas, suggesting that reducing the disease burden requires a combination of community- and person-driven TB initiatives.

## Introduction

The public health issue of tuberculosis (TB) persists despite being treatable and preventable ((1)). Tuberculosis in children younger than 15 years of age, also referred to as paediatric TB, has historically received little attention ((2–4)). Several studies have suggested that the true paediatric TB burden has been incorrectly estimated because a higher proportion of extra-pulmonary TB patients are not reported ((2,5)). Recent statistics indicate that in 2022, children younger than 15 years of age accounted for 12% of all TB cases reported worldwide ((6)).

In Canada, 7% of all reported TB cases in 2022 were paediatric ((7)). This statistic underscores the relatively lower incidence of TB in children within the national context, contrasting with higher proportions observed in specific subpopulations. For example, in Canada, children of Indigenous descent constituted 61% of all paediatric TB cases in 2019. Within this group, First Nations children specifically represented 25% of the total paediatric TB cases ((3)). In Saskatchewan, First Nations children younger than 15 years of age comprised 21% of all active TB cases in 2020 ((8)). Paediatric TB has shown an increasing trend within Saskatchewan’s First Nations communities ((9)). By 2022, 45% of active TB cases in these communities were younger than 15 years of age, reflecting a 74% rise compared to 2021 ((9)).

Moreover, research shows that paediatric TB infections are challenging to recognize early and require immediate care since they have a higher risk of severe outcomes ((1,3,4,10)). Understanding the epidemiology of paediatric TB and the effects of current TB control methods is crucial for addressing the difficulties. This is particularly critical given the discontinuation of Bacille Calmette-Guérin (BCG) vaccination, a key component of the TB elimination strategy for infants in high TB incidence areas in Canada ((11)). Notably, routine BCG vaccination was discontinued among on-reserve First Nations infants in high-TB incidence communities in northern Saskatchewan in September 2011 ((12)).

Few studies ((13,14)) have examined paediatric TB in Canadian First Nations communities and there are still gaps in our understanding of how clinical and socioeconomic factors affect the current paediatric TB epidemic among northern Saskatchewan First Nations on-reserve. Our literature review identified a gap in research specifically addressing paediatric TB in First Nations communities in northern Saskatchewan, despite reported TB outbreaks in the region ((15,16)). This underscores the urgent need to assess and understand the paediatric TB situation in this vulnerable population to tailor appropriate interventions suited to local circumstances. Thus, our study aimed to provide an epidemiological description of paediatric TB among on-reserve First Nations communities in northern Saskatchewan.

## Methods

### Study population and sites

**Figure 1:**
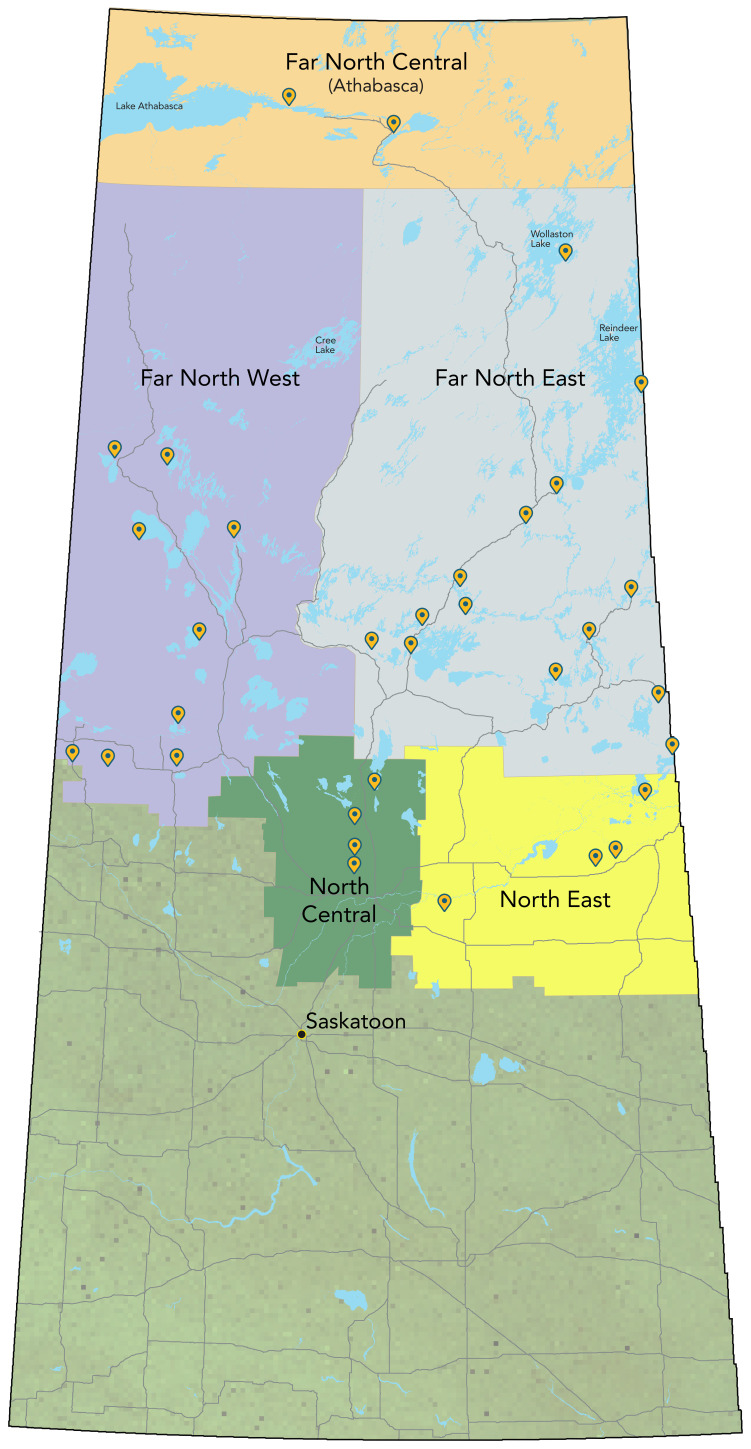
Map of geographic zones^a^ of northern on-reserve Saskatchewan First Nations communities ^a^ Five geographic zones: Far North Central depicted in peach, Far North West in purple, Far North East in grey, North East in yellow and North Central in green

Our study was carried out in First Nations communities in northern Saskatchewan. In the region, there are 33 First Nation communities situated on reserves, collectively housing approximately 55,000 residents, with close to one-quarter of them younger than 15 years of age ((17)). **Figure 1** illustrates these communities categorized into five geographic zones: Far North Central, Far North West, Far North East, North East and North Central. The on-reserve First Nations communities within these geographic zones fall under the jurisdiction of the Northern Inter-Tribal Health Authority (NITHA). This organization collaborates closely with Community Bands and Tribal Councils to deliver a comprehensive range of public health services, aiming to enhance the health and well-being of the First Nations population. These services encompass communicable disease control, immunization, specialized program support, research initiatives, ongoing health status monitoring, training programs, disease surveillance and other technical assistance ((17)).

The study population was restricted to and included all those younger than 15 years of age with a clinical diagnosis of TB or laboratory-confirmed results in the study area ((10,18)). Clinical diagnosis relied on a positive tuberculin skin test (TST) or interferon-gamma release assay (IGRA), abnormal chest x-ray, contact history and clinical symptoms including prolonged fever, persistent cough and failure to thrive ((3)). The positive results of sputum Acid-Fast Bacilli (AFB) smear microscopy and the culture for confirmation were used to make the laboratory diagnosis ((3,15,19)).

### Data collection

We analysed the epidemiological trend and characteristics for paediatric TB in the study group. De-identified individual-level demographic and clinical data of reported confirmed paediatric TB cases from 2018 and 2022 were extracted from the NITHA TB surveillance database. The database is a comprehensive repository that serves as a crucial resource, systematically documenting epidemiological information and clinical profiles related to TB cases within First Nations communities in northern Saskatchewan. The utilization of this database ensures rigorous data integrity and facilitates in-depth analyses essential for understanding and addressing TB challenges in this specific population. The community-level data used in this study were from the 2016 First Nations Community Well-Being index statistics ((20)). Based on the 2016 Census of Canada, Indigenous Services Canada created the publicly accessible Community Well-Being estimates used in this study ((20)).

### Study variables

The demographic factors at the individual level that were examined included the client’s age, sex and geographic zone (**Table 1**). The clinical parameters that were taken into account in this study were case detection year, TB history, disease site, method of detection, BCG vaccination, clinical outcomes and treatment status. According to the healthcare provider’s treatment audit, TB treatment regimens administered under Directly Observed Therapy that were successfully completed were deemed to be treated in our study. In contrast, those still receiving treatment were considered to be on treatment. Those who did not finish their TB therapy but passed away while receiving it were considered to have died during treatment. In our study, we used community-level metrics such as housing score, education score and income score, which varied from zero to 100 ((21)). The adequacy of housing, based on the percentage of a community’s population residing in homes that are not overcrowded and do not require major repairs, is referred to as the housing score (adequate housing level; Table 1). The percentage of a community’s population with a high school diploma or higher was used to calculate the education score (community education level; Table 1). The percentage of the community’s per capita income was used to compute the income score (community income level; Table 1) ((21)). Based on each of these community-level factors, communities were classified as low (less than 50 points) or high (50 or more points) ((21)).

**Table 1:** Summary of study variables

Variable name	Variable description	Variable classification
**Outcome variable**		
Active TB	Children younger than 15 years of age diagnosed with active TB	Counts
**Individual-level variables**		
Age	Child’s age at TB diagnosis	Categorical; 0–4 years, 5–9 years, 10–14 years
Sex	Sex of the paediatric active TB client at birth	Categorical; male, female
Geographic zone	Geographic location of participants by zone	Categorical; Far North Central, Far North East, Far North West, North East
Year	Year the TB case was diagnosed	Categorical; 2018, 2019, 2020, 2021, 2022
Prior history of TB	Previous active or latent TB infection	Categorical; yes, no
Disease site	Location of TB infection	Categorical; pulmonary, disseminated, lymphatic/meningitis
Method of detection	How active TB was identified	Categorical; contact investigation, symptomatic, screening
BCG vaccination	Whether BCG was received	Categorical; yes, no, unknown
Treatment status	Current TB treatment state	Categorical; completed treatment, on treatment, died during treatment
Hospitalization	Ever had TB related hospital admissions	Categorical; yes, no
**Community-level variables**		
Adequate housing level	Proportion of a community’s residents who live in uncrowded, reasonably maintained homes	Categorical; high (50 or more points) or low (less than 50 points)
Community education level	Percentage of a community’s residents with a high school diploma or higher	Categorical; high (50 or more points) or low (less than 50 points)
Community income level	Community’s income per capita expressed as a percentage	Categorical; high (50 or more points) or low (less than 50 points)

Abbreviations: BCG; Bacille Calmette-Guérin; TB, tuberculosis

### Data analysis

Descriptive statistical analyses were carried out utilizing TB data from northern Saskatchewan First Nations communities. The frequency and percentage of paediatric TB cases were computed and tabulated based on both individual and community-level variables. The annual paediatric TB incidence per 100,000 children younger than 15 years old for the research period was calculated. To estimate the TB incidence, we divided the number of new paediatric TB cases that occurred during the specified time period by the total study population at risk (children younger than 15 years of age) multiplied by 100,000 children. Further, age- and sex-based paediatric TB incidence rates per 100,000 children were estimated for each year during the study period. All statistical data analyses were carried out using STATA version 17.0 (StataCorp LLC, Texas, United States). Line graphs displaying paediatric TB incidence were created with Microsoft Excel version 2021(Microsoft Corporation, Washington, United States).

## Results

Overall, we identified 60 paediatric TB cases among children younger than 15 years of age between 2018 and 2022 in northern Saskatchewan First Nations on-reserve communities. The data showed a significant upward trend in the reported cases: there were four cases (7%) in 2018, increasing to six cases (10%) in both 2019 and 2020, 16 cases (27%) in 2021 and 28 cases (47%) in 2022. **Table 2** further showed that among paediatric TB cases, children younger than the age of five made up the majority (55%) of the cases, followed by those between the ages of five and nine years (35%) and 10 and 14 years (10%). According to Table 2, 60% of paediatric TB cases were male and 40% were female. Another aspect of this study was geographical variation. Forty-seven percent of paediatric TB cases lived in the Far North East zone, while 43% were in the Far North Central zone. The remaining paediatric TB clients were located in the Far North West (8%) and North East (2%) zones.

**Table 2:** Distribution of active paediatric tuberculosis cases by demographic characteristics in northern Saskatchewan First Nations communities, 2018–2022

Demographic characteristics	Active paediatric TB cases	
	Total number of cases (n=60)	Percentage
**Year**		
2018	4	7%
2019	6	10%
2020	6	10%
2021	16	27%
2022	28	47%
**Age group (years)**		
0–4	33	55%
5–9	21	35%
10–14	6	10%
**Sex**		
Male	36	60%
Female	24	40%
**Geographic zone**		
Far North Central	26	43%
Far North East	28	47%
Far North West	5	8%
North Central	0	0%
North East	1	2%

Abbreviation: TB, tuberculosis

Over a five-year period, the average paediatric TB incidence was 112.6 cases per 100,000 children each year. The paediatric TB incidence in children aged 0–4 years (277.6 cases per 100,000 children) was also greater than that in children aged 5–9 years (103.7 cases per 100,000 children) and in children aged 10–14 years (28.4 cases per 100,000 children). Males (132.7 cases per 100,000 children) had a higher average annual paediatric TB incidence during the study period compared to females (91.8 cases per 100,000 children). The Far North Central region had the greatest average annual incidence of paediatric TB (696.1 cases per 100,000 children), followed by the Far North East (116.8 cases per 100,000 children), the Far North East (47.0 cases per 100,000 children) and the North East (11.4 cases per 100,000 children) (**Table 3**).

**Table 3:** Distribution of active paediatric tuberculosis and incidence by demographic characteristics in northern Saskatchewan First Nations communities, 2018–2022

Demographic characteristics	Average annual paediatric TB cases (n)	Population of children under 15 years (N)	Average paediatric TB incidence per year (per 100,000 children)
Total	12	10,653	112.6
**Age group (years)**			
0–4	6.6	2,377	277.6
5–9	4.2	4,051	103.7
10–14	1.2	4,226	28.4
**Sex**			
Male	7.2	5,227	132.7
Female	4.8	5,426	91.8
**Geographic zone**			
Far North Central	5.2	747	696.1
Far North East	5.6	4,794	116.8
Far North West	1.0	2,130	47.0
North Central	0.0	1,229	0.0
North East	0.2	1,753	11.4

Abbreviation: TB, tuberculosis

**Figure 2:**
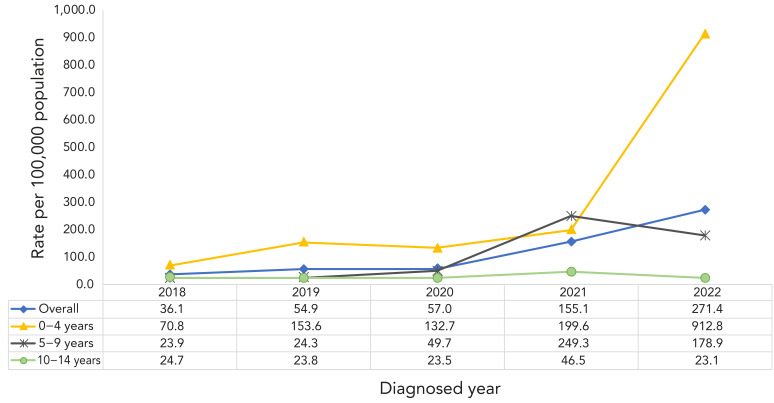
Active paediatric tuberculosis incidence by age group in northern Saskatchewan First Nations on-reserve communities, 2018–2022

The active paediatric TB incidence increased by 644.0% from 36.1 cases per 100,000 children in 2018 to 268.6 cases per 100,000 children in 2022 (**Figure 2**). Between 2018 and 2022, the paediatric TB incidence in the 0–4 year age group (from 70.8 cases per 100,000 children to 912.8 cases per 100,000 children) and the 5–9 year age group (from 23.9 cases per 100,000 children to 178.9 cases per 100,000 children) increased by 1,189.3% and 648.5%, respectively. The paediatric TB incidence in children aged 10–14 years declined by 6%, from 24.7 cases per 100,000 children in 2018 to 23.1 cases per 100,000 children in 2022 (Figure 2).

**Figure 3:**
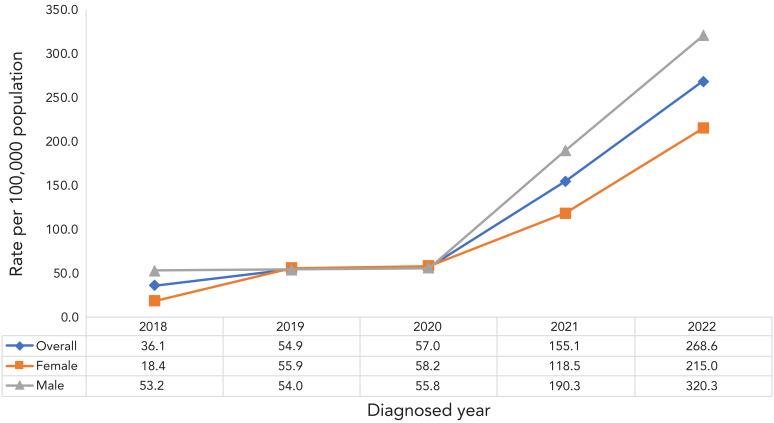
Active paediatric tuberculosis incidence by sex in Northern Saskatchewan First Nations on-reserve communities, 2018–2022

Although the paediatric TB rates in both sexes showed an upward trend over the study period between 2018 and 2022 in the northern Saskatchewan First Nations communities, the percentage annual change was higher among females (**Figure 3**). The paediatric TB rate in females increased by 1,068%, from 18.4 cases per 100,000 children in 2018 to 215.0 cases per 100,000 children in 2022, whereas male paediatric TB rate increased by 502%, from 53.2 cases per 100,000 children in 2018 to 320.3 cases per 100,000 children in 2022.

The majority of paediatric TB cases, 58 of 60 cases (97%), had no history of prior TB infection (**Table 4**). The majority (85%) of paediatric TB clients were pulmonary TB. Disseminated TB (8%) and lymphatic or meningitis TB (7%) accounted for a relatively small number of paediatric TB cases. Additionally, 85% of paediatric TB cases were detected through contact investigations as opposed to 13% and 2% of paediatric TB cases identified by symptomatic and screening investigations, respectively (Table 4). Given that BCG has not been used since 2011 among northern Saskatchewan First Nations communities on reserves, only 3% of participants self-reported having received a BCG vaccination, compared to 90% of those who had no BCG documentation.

**Table 4:** Distribution of paediatric tuberculosis cases by clinical characteristics in Northern Saskatchewan First Nations on-reserve communities, 2018–2022

Clinical characteristics	Active paediatric TB cases	
	Number of cases (n=60)	Percentage
**Prior history of TB**		
Yes	2	3%
No	58	97%
**Disease site**		
Pulmonary	51	85%
Disseminated	5	8%
Lymphatic/meningitis	4	7%
**Method of detection**		
Contact investigation	51	85%
Symptomatic	8	13%
Screening	1	2%
**BCG vaccination**		
No	4	7%
Yes	2	3%
Unknown	54	90%
**Treatment status**		
Completed	43	71%
On treatment	16	27%
Died during treatment	1	2%
**Hospitalizations**		
Ever hospitalized	15	25%
No admissions	45	75%

Abbreviations: BCG, Bacille Calmette-Guérin; TB, tuberculosis

At the end of the study period, 27% of paediatric TB cases were still receiving treatment, 71% had successfully finished Directly Observed Therapy and 2% had died while receiving treatment. Only one-quarter (25%) of paediatric TB cases have ever been admitted to the hospital for tuberculosis-related reasons (Table 4).

**Table 5** presents the distribution of active paediatric TB cases across various community characteristics. The analysis reveals significant disparities across various socioeconomic factors. Communities with a high level of adequate housing reported 20 cases (33%), whereas those with a low level had 40 cases (67%). Similarly, the income level analysis showed that communities with high income levels had 20 cases (33%), while those with low-income levels had 40 cases (67%). Regarding education, all cases (100%) occurred in communities with low education levels.

**Table 5:** Distribution of active paediatric tuberculosis cases by community-level characteristics in Northern Saskatchewan First Nations communities, 2018–2022

Community characteristics	Active paediatric TB cases	
	Number of cases (n=60)	Percentage
**Adequate housing level**		
High	20	33%
Low	40	67%
**Community education level**		
High	0	0%
Low	60	100%
**Community income level**		
High	20	33%
Low	40	67%

Abbreviation: TB, tuberculosis

## Discussion

This study was carried out in First Nations on-reserve communities in northern Saskatchewan to shed light on the factors that influence the paediatric TB distribution over time. To deliver context-specific TB care, our analysis identified the characteristics of paediatric TB cases among First Nations children in these communities.

The estimated paediatric TB rate in this study (112.6 cases per 100,000 children) was higher than the paediatric TB rates among all Canadian First Nations children residing on reserves (20.2 cases per 100,000 children) and that of the general population of children in Canada (1.2 cases per 100,000 children) ((18)). The disproportionately higher rate among this study group could be linked to malnutrition, possibly exacerbated by persistent food insecurity prevalent in First Nations communities. This condition increases the susceptibility of children to developing TB following exposure ((14,22)). Prior studies indicate that the increased incidence of paediatric TB could stem from ongoing shortages and frequent turnover among healthcare staff specializing in TB ((14,15,23)). These workforce challenges can result in delays in both diagnosing the disease and initiating treatment ((14,15,23)).

A Canadian study ((18)) reported that 50.5% of paediatric TB cases were male; however, this study found a higher proportion of paediatric TB cases among males (60%). Our study revealed patterns of increased paediatric TB incidence in both sexes, with particularly higher rates among males. Several factors may contribute to this disparity. Previous research has suggested that physiological differences and behavioral patterns between males and females could affect TB susceptibility and progression in males ((24,25)). However, a study reported no significant difference in TB incidence between male and female children under 15 years of age ((26)). Given the varied findings, assessing the overall contribution of sex-specific differences in tuberculosis incidence remains challenging. Future research should prioritize sex-specific investigations into paediatric TB incidence to better understand the underlying factors contributing to the observed disparities.

The majority of paediatric TB cases in this study were among children younger than five years, which is consistent with an earlier study ((14)). The escalating trend of paediatric TB cases among children younger than five years compared to older age cohorts in the study group warrants careful examination. Younger children have shown higher susceptibility to TB due to several factors. Firstly, children younger than five years have developing immune systems, making them more vulnerable to infections including TB ((14,27–29)). Secondly, household transmission dynamics can lead to increased exposure among younger children who are in close contact with infectious adults ((3)). Thirdly, diagnostic challenges such as difficulties in obtaining adequate sputum samples for testing contribute to delayed or missed diagnoses in this age group ((3,4)). The delayed or missed diagnosis can lead to progression of TB infection into life-threatening forms, including disseminated TB and TB meningitis ((14,29)). Beyond diagnostic challenges with obtaining samples as outlined below, it should be noted that children of this young are often asymptomatic or present with vague symptoms and often, their cultures, even if obtained, are of lower yield, as they tend to be paucibacillary. Also, children in this age group are just generally at higher risk for higher morbidity and mortality with TB disease progression.

The total number of paediatric TB cases in Saskatchewan’s northern First Nations population living on reserves over the study period were reported in four different geographic areas. Most of the paediatric TB cases in this study were reported in the Far North Central and Far North Eastern regions; perhaps the communicability of TB and location may have influenced the TB incidence of as suggested in other studies ((18,30)). The Northern Saskatchewan First Nations TB Program relies on the expertise of TB nurses, community health nurses, lay TB workers and a medical health officer to provide timely, safe and competent TB care. However, the program’s effectiveness maybe hindered by inadequate staffing and challenges in accessing healthcare in remote First Nations settings ((17)). Similar studies indicate a connection between geographical discrepancy and a shortage of TB healthcare experts, difficulties with patient transportation and logistics ((15,30)). The disparity in paediatric TB incidence between geographic areas may also be explained by community social networks that increase susceptibility to TB infection and challenging obstacles to seeking and pursuing TB care ((28)). Furthermore, the remoteness of communities may exacerbate issues including access to healthcare and early TB diagnosis and treatment, as suggested in previous studies ((14,30)).

Most paediatric TB cases in our study were pulmonary TB, which is consistent with other studies ((18)). This is possibly because of the immune system weakness that has been linked to TB predisposition in children, as described in prior studies ((31,32)). Similar to our analysis, a substantial number of the paediatric TB cases required hospitalization ((18,33)). These hospitalizations are likely due to the challenges in identifying TB symptoms in young children, who often present with nonspecific clinical signs. Such challenges can lead to diagnostic delays and potentially exacerbate disease outcomes ((3)).

Similar to prior studies conducted in Canada, our study demonstrates that contact investigations uncovered the majority of paediatric TB cases living in northern Saskatchewan First Nations on-reserve communities ((15,18)). In order to further improve contact investigation, it is necessary to overcome challenges such perceived TB stigma, understaffed TB workers and contacts’ poor TB knowledge ((28,32–37)).

Additionally, our study provided evidence to support the fact that living conditions are subpar on reserves. According to previous research, inadequate housing, low rates of higher education and low-income level all contribute to the persistence of TB transmission ((30,38)). In our study, paediatric TB cases were stratified by community-level characteristics and disparities were examined similar to a prior study ((38)). The level of overcrowded and inadequate housing in the community may have affected the frequency of paediatric TB clients. Our study found that people from First Nations on-reserve communities with lower adequate housing had the highest occurrence of paediatric TB cases. Our findings are consistent with past studies that emphasized the important role that homelessness and crowded and/or poorly maintained dwellings play in the transmission of TB ((15,29,30,39)). Given the high rates of substandard housing and overcrowding, which were identified in First Nations on-reserve communities in a prior study, this was expected ((30,40)). A comparable study has observed the impact of family structure and culture on large households ((41–43)), and this may play a role given that First Nations People on-reserve often have large families and therefore more children living in relatively small dwellings ((44)).

The findings of our study are consistent with other research in that people who live in communities with higher levels of education are probably less likely to experience paediatric TB incidences ((45)). The trauma experienced in residential schools may account for the low community education levels, as documented in a previous study ((14)). Communities with higher percentages of individuals possessing advanced education may exhibit greater knowledge about the causes, risk factors, symptoms and treatments of TB. This enhanced awareness can influence one’s frequency of seeking medical assistance and adherence to TB prevention measures ((45)).

Finally, the results of our study supported prior research ((30)) that suggested a connection between community income level and the incidence of TB, showing that paediatric TB cases were more common among residents of lower-income communities. This study’s findings are consistent with notions that TB is a social sickness, with major medical repercussions, that is fueled by poverty ((14)). A lower degree of community income can lead to more TB cases by resulting in food insecurity and impeding access to health care through transportation cost, as well as other related economic costs ((14)).

### Study strengths and limitations

This study used high-quality data to address the local TB context and epidemiology in First Nations communities. We evaluated the trend of paediatric TB over a five-year period for the first time among First Nations communities in northern on-reserve Saskatchewan communities. It becomes increasingly challenging to identify, stop and eventually eradicate TB among individuals who are most at risk. Perhaps these challenges are a result of the dearth of current, reliable and trustworthy information regarding the background risk of paediatric TB among First Nations peoples at the community level ((23)).

Due to a lack of available data, we excluded certain variables from our study. For instance, prior research has linked cultural factors, historical colonial trauma and food instability to the persistence of TB transmission ((14,30,44)) but these factors were not taken into account in our study due to lack of data. More research is required to promote culturally acceptable TB care practices that respect cultural diversity and foster an inclusive atmosphere in First Nations communities. Future studies should employ rigorous analytical methods to mitigate the limitations in establishing causal relationships or pathways observed in this study. The generalizability of our findings may be constrained by the specific context of the study population. Additionally, the dichotomous nature of the Community Well-being Index data used in our study might restrict nuanced interpretations of community conditions.

### Public health implications

The evidence from this study suggests that First Nations communities in northern Saskatchewan are experiencing an increase in paediatric TB cases. To improve paediatric TB control and care in northern Saskatchewan First Nations communities, public health professionals will potentially benefit from the findings of our study in terms of its implication on risk factors and contact tracing investigations. According to past studies, the insight from this study can aid in the rapid identification of paediatric TB, which may lower the severity of the patient’s illness and possibly halt widespread paediatric TB infections among households and within the community ((46)).

Despite these steps, an earlier study viewed them as short-term solutions for stopping TB transmission within the northern Saskatchewan First Nations population ((47)). If eradication is the long-term objective, then dealing with the socioeconomic problems identified in this study, poverty, inadequate housing and education, that have contributed to the spread of TB is imperative ((14)). As indicated in an earlier study, more housing must be built and current housing must be repaired in order to address these concerns ((48)). Additionally, as revealed in a prior study, boosting food and other incentive programs may help combat the spread of TB in low-income communities ((48,49)). Promoting higher education and increasing TB awareness, as proposed by a previous study, will help to minimize stigma and discrimination ((14,50,51)). These efforts may increase the uptake of TB care and preventive services in northern Saskatchewan First Nations communities.

## Conclusion

Paediatric TB continues to disproportionately impact First Nations communities in northern Saskatchewan, a gap that may be mostly attributable to social determinants of health. Four of the five geographical zones in this study exhibited a significant burden of paediatric TB cases. This study found that paediatric TB rates were higher in males than in females and highest in children younger than five years. This study emphasizes the critical need to successfully address the long-standing socioeconomic problems in the community, like poverty, inadequate housing and inadequate education, which significantly contribute to the spread of TB. It also highlights the importance of contact investigation in the early detection of new paediatric TB infections. This research demonstrates that combining community-based and individual-focused TB initiatives can lead to substantial progress.
